# Efficacy and safety of variceal embolization for primary prophylaxis in cirrhosis patients with challenges in standard treatments: preliminary results

**DOI:** 10.3389/fmed.2024.1401900

**Published:** 2024-07-15

**Authors:** Jun Tie, Xulong Yuan, Ying Zhu, Kai Li, Xiaoyuan Gou, Na Han, Jing Niu, Jiao Xu, Wenlan Wang, Yongquan Shi

**Affiliations:** ^1^State Key Laboratory of Holistic Integrative Management of Gastrointestinal Cancers, National Clinical Research Center for Digestive Diseases and Xijing Hospital of Digestive Diseases, Air Force Medical University, Xi'an, Shaanxi, China; ^2^Department of Aerospace Hygiene, School of Aerospace Medicine, Air Force Medical University, Xi'an, Shaanxi, China

**Keywords:** variceal embolization, primary prophylaxis, cirrhosis, hypertension, portal, variceal bleeding

## Abstract

**Objectives:**

Nonselective beta blockers (NSBBs) or endoscopic therapies are currently recommended by guidelines for preventing the first variceal bleed in patients with high-risk varices. However, there is a lack of detailed treatment strategies for patients who are intolerant to both NSBBs and endoscopic approaches. Our study aimed to assess the efficacy and safety of variceal embolization as a primary prophylaxis method in cirrhosis patients who are not suitable candidates for NSBBs or endoscopic treatments.

**Methods:**

The study included 43 cirrhotic patients with high-risk varices who were candidates for primary prophylaxis against variceal bleeding. These patients underwent variceal embolization at the Xijing Hospital between January 2020 and June 2022. The primary endpoint was the occurrence of bleeding from varices, and the secondary endpoints were the recurrence of varices and the emergence of complications.

**Results:**

The procedure of variceal embolization had a success rate of 93.0% (40 out of 43 patients). Over a 2-year follow-up period, the rate of variceal bleeding was 11.6% (5 out of 43 patients), the recurrence rate of varices was 14.0% (6 out of 43 patients), and the rate of severe complications was limited to 2.3% (1 out of 43 patients).

**Conclusion:**

Variceal embolization is a viable primary prophylactic intervention for cirrhotic patients who are at risk of variceal bleeding when standard treatments, such as NSBBs or endoscopic therapies, are difficult to perform.

## Introduction

Variceal bleeding is a serious complication of portal hypertension secondary to cirrhosis. At the point of diagnosis, approximately 50% of patients with cirrhosis are found to have esophageal and/or gastric varices (EGVs). Patients without EGV at diagnosis develop varices at an annual rate of approximately 8%, and patients with small EGV pose a 22% annual risk of progression to medium or large varices. Additionally, 10–15% of patients with EGV experience their initial variceal bleeding each year, and the mortality rate for the first episode of variceal bleeding is high, with approximately 15% of patients dying from this initial bleeding (1, 2). Therefore, primary prophylaxis to prevent the first episode of variceal bleeding is essential in the management of cirrhosis.

Current guidelines recommend non-selective beta-blockers (NSBBs) as the first-line treatment for preventing the first variceal hemorrhage in patients with high-risk EGV. If NSBBs are contraindicated or not tolerated, endoscopic band ligation (EBL) is the preferred alternative for esophageal varices (EVs). For high-risk GVs, endoscopic cyanoacrylate injection (ECI) is recommended when NSBBs are not suitable (3–6). However, the guidelines currently lack detailed treatment strategies for patients who are unable to tolerate both NSBBs and endoscopic procedures.

Variceal embolization encompasses a range of interventional procedures aimed at occluding varicose veins. This approach is minimally invasive, technically straightforward, and has been confirmed to have efficacy and safety in both acute variceal bleeding treatment and secondary prophylaxis (7–9). Notably, in the treatment of GV, embolization with cyanoacrylate is superior to ECI (10). Given the efficacy and safety profile of variceal embolization, we hypothesize that it could also be a viable option for primary prophylaxis against variceal bleeding. This study aims to assess the effectiveness and safety of variceal embolization as a primary prophylactic intervention in cirrhosis patients who are not candidates for NSBBs and endoscopic treatments.

## Methods

### Study design

This was a single-arm retrospective observational study. It consecutively enrolled patients with cirrhosis and high-risk EGV who were admitted to Xijing Hospital from January 2020 to June 2022. Follow-up for the last patient extended beyond 6 months. Written informed consent was obtained from all patients for the use of their data in this research. The study was approved by the Medical Ethics Committee of the First Affiliated Hospital of Air Force Medical University (approval number: KY20232008-C-1).

The inclusion criteria were as follows: (1) patients who are aged between 18 and 75 years; (2) patients with cirrhosis diagnosed by biopsy or a combination of medical history, etiology, physical examination, clinical presentation, biochemical testing, and imaging; (3) patients with high-risk EGV confirmed by endoscopic examination; (4) patients difficult to be treated with NSBBs and EBL/ECI as evaluated by two endoscopists; and (5) patients receiving variceal embolization with cyanoacrylate and/or coil for the prevention of EGV bleed. The exclusion criteria were as follows: patients with (1) a history of variceal bleeding; (2) hepatocellular carcinoma (HCC) beyond the Milan criteria; (3) malignant tumors excluding HCC; (4) main portal vein thrombus of over 50% or cavernous transformation of the portal vein; (5) recurrent or refractory ascites; (6) end-stage renal disease with renal replacement treatment; (7) cardiorespiratory failure; (8) human immunodeficiency virus infection or acquired immune deficiency syndrome; or (9) lack of baseline data and/or follow-up data.

### Data collection and follow-up

The following data were collected for all patients: (1) baseline data including age, sex, etiology of liver disease, liver function, variceal type, and enhanced CT/MRI; (2) treatment information including access to the operation, embolization details, and operation-related complications; and (3) follow-up data including main symptoms, variceal bleeding, blood biochemistry, the changes of varices, and overall survival.

Endoscopic evaluations of varices were performed at 1, 3, and 6 months after the initial procedure and subsequently at 6-month intervals to monitor variceal changes. The study was concluded for each patient either upon the occurrence of an endpoint event, which was either variceal bleeding or recurrence, or after a minimum follow-up duration of 6 months post-embolization.

### Variceal embolization with cyanoacrylate and steel coils

Our study utilized two approaches to variceal embolization: percutaneous transhepatic varices embolization (PTVE) and transjugular varices embolization (TJVE). PTVE involves accessing the varices via a percutaneous transhepatic route, known for its ease of operation. TJVE, alternatively, uses a transjugular intrahepatic route, which is preferred in patients with severe thrombocytopenia or significant ascites due to its reduced risk of abdominal hemorrhage. The criteria for selecting the approach were based on platelet counts and the presence of ascites: PTVE for patients with platelet counts above 50 × 10^3^/μL without ascites and TJVE for those with counts below 50× 10^3^/μL or with significant ascites.

**Figure 1:**
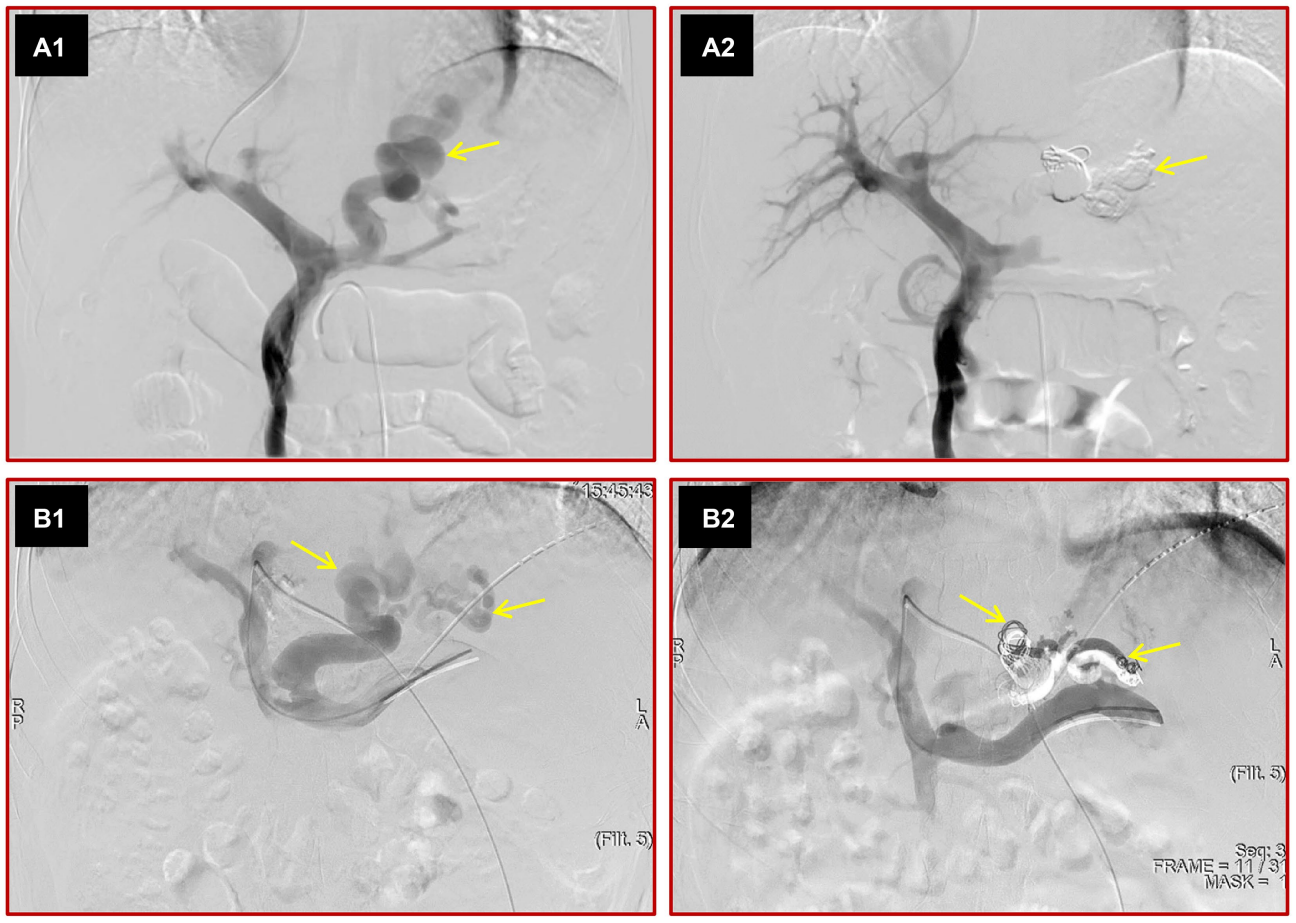
Images depict the two kinds of variceal embolization methods. The portal vein was punctured from the hepatic vein through internal jugular vein access, direct portal vein angiography was performed, and large varices (yellow arrow) were seen **(A1)**. After variceal embolization with cyanoacrylate and steel coils, the varices disappeared on direct portal angiography **(A2)**. The left portal vein was punctured by a percutaneous transhepatic approach, direct portal vein angiography was performed, and various large varices (yellow arrows) were observed **(B1)**. After variceal embolization with cyanoacrylate and steel coils, the varices were not seen **(B2)**.

The procedures were performed by Dr. Tie Jun, who possesses extensive experience with over a decade in conducting transjugular intrahepatic portosystemic shunt (TIPS) operations and has a professional record of conducting over 1,000 procedures. The TJVE procedure was conducted as follows: Initially, the femoral artery was accessed using the Seldinger technique, and then the superior mesenteric artery was selectively intubated for indirect portal vein angiography. Following this, the internal jugular vein was punctured, and a 0.035-inch guide wire (Terumo, Tokyo, Japan) was introduced through it into the inferior vena cava. Over this guide wire, the RUPS 100 puncture set (Cook, Chicago, United States) was advanced into the inferior vena cava. Subsequently, a portal vein branch was accessed from the right hepatic vein or the inferior vena cava, with placement confirmed by pushing a small amount of contrast agent. The guide wire was then maneuvered to the distal segment of the splenic or superior mesenteric vein for direct portal vein angiography, which facilitated the assessment of the varices in terms of location, morphology, size, and blood flow velocity. The 5F angiographic catheter was used to selectively intubate the varices. The varices were embolized with steel coils of appropriate diameter until the blood flow velocity of the varices slowed significantly. Then, a coaxial microcatheter (Boston Scientific Corporation, Massachusetts, United States) was inserted into the varices. The mixture of cyanoacrylate and iodide oil was slowly injected to make them flow slowly along the varicose veins and distribute in the reticular branches of the distal varicose veins until they were completely embolized. The ratio of iodide oil to cyanoacrylate was 3:1. For varicose veins with diameters greater than 10 mm, the ratio of iodide oil to cyanoacrylate was reduced. After the embolization was completed, the end of the angiography catheter was placed at the splenic hilum, and direct portal vein angiography was performed again to confirm whether the varicose veins were completely embolized (Figure 1A).

The main PTVE procedure was as follows. The intrahepatic portal vein branch was punctured under ultrasound guidance. The guide wire was adjusted to the splenic vein or distal superior mesenteric vein, and the 5F angiographic catheter was advanced over the guide wire. Then, direct portal vein angiography was performed. The variceal embolization process was the same as that for TJVE. After variceal embolization, the puncture path was sealed with steel coils and cyanoacrylate (Figure 1B).

### Definitions

High-risk EGV: grade I varices and red signs, grade 2–3 varices, and GVs larger than 10 mm in diameter (4, 5). EVs type 1 refers to grade I varices with positive red signs. EVs type 2 refers to grade 2–3 varices with or without positive red signs.

The types of high-risk EGVs are EV and GV. The GV was classified into gastroesophageal varices 1 (GOV1), gastroesophageal varices 2 (GOV2), isolated GV 1 (IGV1), and isolated GV 2 (IGV2), according to the Sarin classification (11).

Variceal bleeding: the presence of medium or large varices and red signs or active bleeding; the presence of blood in the stomach without any cause other than large varices (12, 13).

Variceal eradication: complete disappearance of varices or the presence of varices smaller than 5 mm in diameter and no red signs (14).

Variceal recurrence: the presence of varices larger than 5 mm in diameter after initial eradication (14).

**Figure 2:**
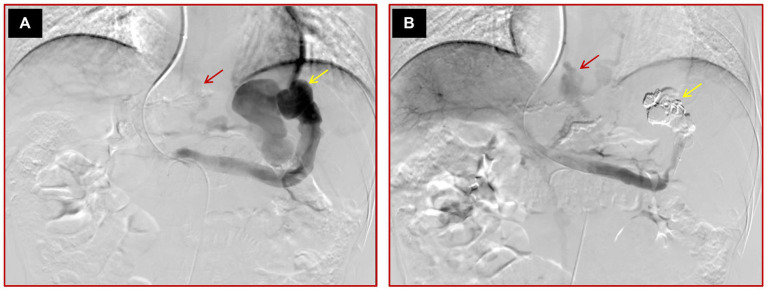
Images of a 45-year-old male cirrhotic patient with high-risk EV and IGV1 who failed variceal embolization. **(A)** The initial direct portal vein angiography reveals multiple varices and a prominent spontaneous splenorenal shunt, highlighted by yellow arrows. **(B)** Post-embolization angiography demonstrates that while the conspicuous spontaneous splenorenal shunt is no longer detectable, signifying partial procedural success, some varices persist, as indicated by a red arrow, suggesting incomplete occlusion.

Successful variceal embolization: direct portal angiography without visible varices after the operation and endoscopic variceal eradication at 1 month after variceal embolization (Figure 2).

Ascites occurrence: the new onset of any detectable ascites post-procedure in patients with no prior history of ascites before undergoing variceal embolization.

Worsening of ascites/hydrothorax: an increase in the ascites/hydrothorax volume from minimal to moderate or large, as determined by imaging or physical examination post-procedure.

### Statistical analyses

All continuous variables are expressed as the median (range). Categorical variables are expressed as counts and percentages.

## Results

### Patient characteristics

**Figure 3:**
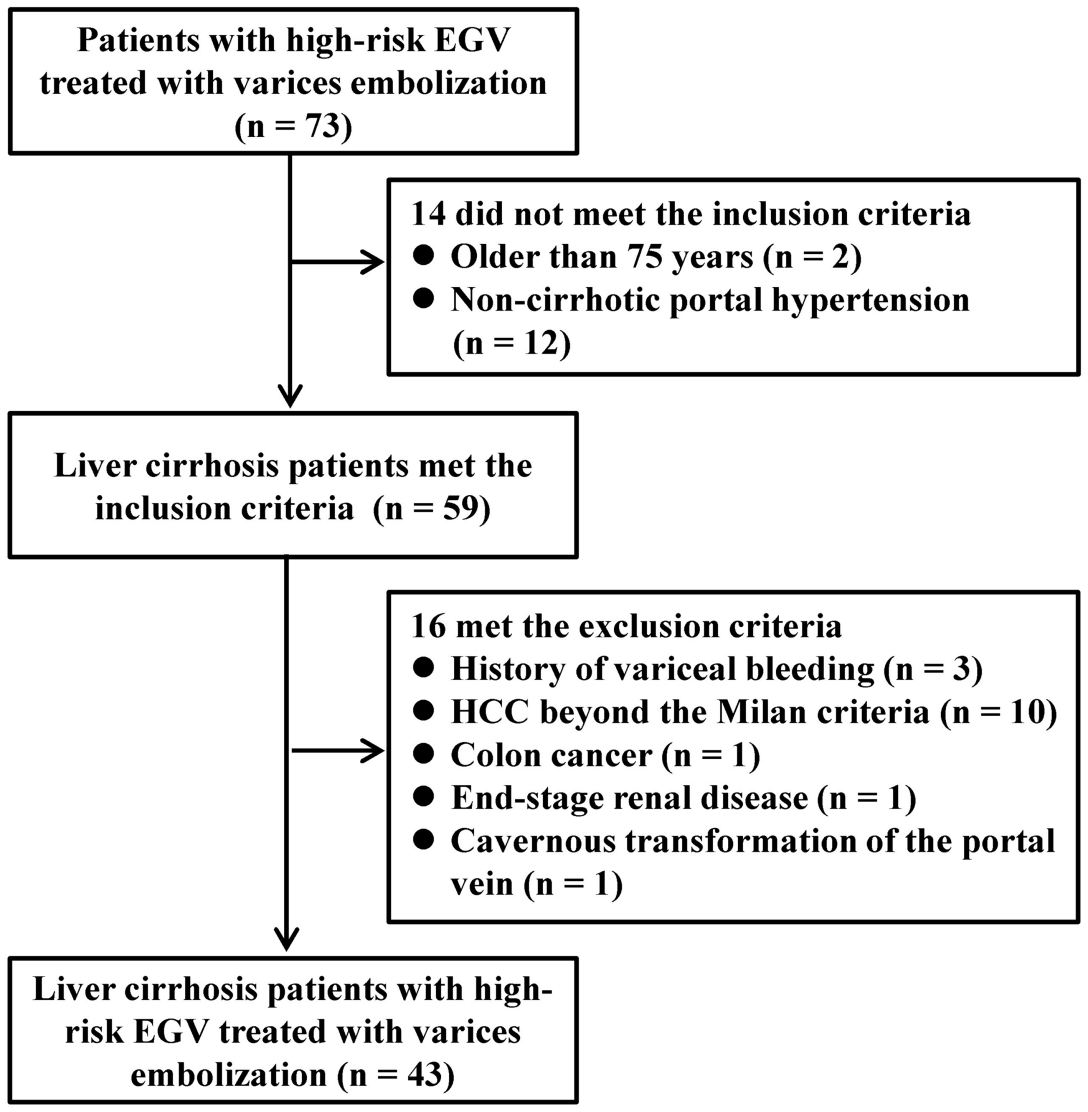
Flowchart of patient selection.

Between January 2020 and June 2022, a total of 73 consecutive patients underwent variceal embolization for high-risk EGV. However, 14 patients were excluded based on the exclusion criteria; 2 were over the age of 75 years, and 12 suffered from non-cirrhotic portal hypertension. An additional 16 patients were excluded for various reasons: 3 had a prior history of variceal bleeding, 10 patients had HCC who did not meet the Milan criteria, 1 was diagnosed with colon cancer, 1 was undergoing renal replacement therapy for end-stage renal disease, and 1 had a cavernous transformation of the portal vein. Consequently, 43 patients were eligible and enrolled in the study (Figure 3). The specific reasons for patients being unsuitable for NSBBs and endoscopic treatments are detailed in Table 1.

**Table 1:** Detailed reasons for exclusion from NSBBs and endoscopic treatment.

Reasons for exclusion from NSBBs	Number (*n* = 43)
History of bronchospasm	13
Second or third-degree atrioventricular block	2
Sick sinus syndrome	2
Resting heart rate below 50 beats per minute.	4
Heart failure or clinically significant hypotension	3
Peripheral vascular disease	1
Liver function classified as Child–Pugh class C	3
History of diabetic ketoacidosis	7
Significant adverse reactions to carvedilol	8
Reasons for exclusion from endoscopic treatment	
Severe coagulopathy	3
Severe thrombocytopenia (<30 × 10^9^/L)	6
Severe cardiopulmonary dysfunction	13
Varices with a diameter greater than 2 cm	21

NSBBs, Non-selective beta-blockers.

Of these participants, the median age was 54 years, with a range from 18 to 74 years. Male patients constituted 44.2% (*n* = 19), and 48.8% (*n* = 21) had hepatitis B virus-related cirrhosis. The median Child–Pugh score was 6, ranging from 5 to 12, and the median Model for End-Stage Liver Disease (MELD) score was 10, ranging from 4 to 22. Regarding varices types, 23.3% (*n* = 10) of the patients exhibited EVs, while 76.7% (*n* = 33) displayed GVs. Among the GV subgroup, 25.6% (*n* = 11) had GOV1, 23.3% (*n* = 10) had GOV2, 9.3% (*n* = 4) had IGV1, and 18.6% (*n* = 8) had a combination of more than one variceal type. Treatment modalities were nearly evenly split, with 51.2% (*n* = 22) undergoing transjugular variceal embolization (TJVE) and 48.8% (*n* = 21) receiving percutaneous transhepatic variceal embolization (PTVE). The follow-up period varied among participants, with the shortest being 77 days and the longest extending to 924 days. The median follow-up duration was 573 days. A comprehensive baseline characteristics of the patient cohort are detailed in Table 2.

**Table 2:** Baseline demographic and clinical characteristics.

Parameter	Value
Median (range) or absolute (percentage)	
Age (years)	54 (18–74)
Sex (Male)	19 (44.2%)
Etiology	
HBV	21 (48.8%)
HCV	3 (7.0%)
Others	19 (44.2%)
Liver function	
MELD scores	10 (4–22)
Child–Pugh scores	6 (5–12)
Child–Pugh class A/B/C	25/15/3
Compensation stage	25 (58.1%)
Type of varices	
Esophageal varices	10 (23.3%)
EV1	1 (2.3%)
EV2	9 (20.9%)
Gastric varices	33 (76.7%)
GOV1	11 (25.6%)
GOV2	10 (23.3%)
IGV1	4 (9.3%)
More than one variceal type	8 (18.6%)
Variceal diameter (mm)	15 (4–25)
Presence of a red sign	26 (60.5%)
Gastro-renal shunt	21 (48.8%)
Ascites/hepatic hydrothorax	13 (30.2%)
Hepatic encephalopathy	7 (16.3%)
Access to the operation	
Transjugular	22 (51.2%)
Transhepatic	21 (48.8%)
Median follow-up (days)	573 (77–924)

HBV, hepatitis B virus; HCV, hepatitis C virus; MELD, model of end-stage liver disease; EV1, esophageal varices type 1; EV2, esophageal varices type 2; GOV1, gastroesophageal varices type 1; GOV2, gastroesophageal varices type 2; IGV1, isolated gastric varices type 1.

### Efficacy of variceal embolization

Out of 43 patients, 93.0% (40 patients) achieved successful embolization, as confirmed by the absence of visible varices on post-operative portal angiography. During the follow-up, five patients (11.6%) experienced variceal bleeding; these individuals were subsequently treated with TIPS therapy. The median interval from embolization to bleeding was 40 days, ranging from 3 to 454 days. Notably, bleeding occurred within 6 weeks for the three patients whose embolization was not successful, whereas it occurred at 250 days and 454 days for the two patients with initial successful procedures. This suggests that failed embolization may lead to an early increase in portal vein pressure and subsequent bleeding from residual varices. In contrast, no short-term bleeding was observed in patients with successful embolization. Over 2 years, the variceal bleeding rate remained at 11.6%. Additionally, 14.0% of patients experienced a recurrence of high-risk EGV, with a median time to recurrence of 195 days. Among the 13 patients with pre-existing hepatic ascites or hydrothorax, 30.8% experienced a worsening of these conditions, typically within 43 days. Conversely, 20.0% of patients without initial ascites and hydrothorax developed ascites, generally after a median time of 371 days. The study recorded a 7.0% mortality rate (three patients) during the follow-up period, but no deaths were directly attributed to variceal bleeding as per the data in Table 3.

**Table 3:** Efficacy of variceal embolization.

Parameter	Value
Successful embolization	40 (93.0%)
First variceal bleed	5 (11.6%)
Median time to first bleed	40 (3–454)
2-year bleeding rate	5 (11.6%)
High-risk EGV recurred	6 (14.0%)
Median time to variceal recurrence	195 (97–605)
2-year EGV recurrence rate	6 (14.0%)
Fresh ascites	6 (14.0%)
Median time to ascites occurrence	371 (257–478)
Hepatic ascites/hydrothorax at baseline	13 (30.2%)
Worsening of ascites/hydrothorax	4 (30.8%)
Median time to worsening of ascites	43 (31–57)
Mortality	3 (7.0%)
Bleeding mortality	0 (0.0%)

### Complications associated with variceal embolization

Abdominal pain was the most frequent complication, occurring in 51.2% of cases (22/43). Generally, the pain resolved within a week of symptomatic treatment. Nausea and vomiting were reported in 30.2% (13/43) of patients. Fever was present in 7.0% (3/43), while portal vein thrombosis was noted in one case (2.3%). There was one instance of abdominal hemorrhage (2.3%), which required hepatic artery embolization for management. Subcutaneous hematoma at the puncture site, either in the internal jugular vein or femoral artery, was observed in 4.7% (2/43) of the patients. These hematomas typically resolve with local compression between 2 and 4 weeks post-operation. Ectopic embolization of cyanoacrylate occurred in 4.7% (2/43) of patients due to large spontaneous portosystemic shunts. However, the embolization particles were small, and the scope of embolization was limited, resulting in no clinical symptoms, and consequently, no intervention was required for these cases, as shown in Table 4. There were no fatal complications reported. Overall, the rate of severe complications, such as abdominal hemorrhage, was low, at only 2.3% (1/43).

**Table 4:** Complications.

Complications	Frequency n (%)
Non-operation-related complications	
Abdominal pain	22/43 (51.2%)
Nausea and vomiting	13/43 (30.2%)
Fever	3/43 (7.0%)
PVT	1/43 (2.3%)
Operation-related complications	
Abdominal hemorrhage	1/43 (2.3%)
Subcutaneous hematoma	2/43 (4.7%)
Ectopic embolism	2/43 (4.7%)

## Discussion

Current guidelines recommend NSBBs or EBL/ECI for the prevention of initial variceal bleeding in patients with cirrhosis. However, these options are not viable for patients who are intolerant to both treatments. In this retrospective study, we assessed the effectiveness and safety of variceal embolization as primary prophylaxis in 43 patients with cirrhosis who were difficult to treat using NSBBs and EBL/ECI. The findings indicated a high success rate of 93.0% for variceal embolization. Over 2 years, the rate of variceal bleeding was 11.6%, and the recurrence of EGV was 14.0%. Importantly, there were no fatal complications associated with the procedure. This study is the first to demonstrate that variceal embolization can be a safe and effective primary prophylactic measure against variceal bleeding in patients who are not candidates for NSBBs and EBL/ECI.

This study suggests that variceal embolization is an effective option for the primary prevention of variceal bleeding. For patients on NSBBs, a reduction in the risk of bleeding is only observed in those who exhibit a hepatic venous pressure gradient (HVPG) response. The HVPG response rate to NSBBs in patients with cirrhosis varies between 30 and 60% (15–17), implying that at least approximately 40% of patients may not benefit from NSBB therapy. Comparatively, the long-term variceal bleeding rate within 2 years for patients undergoing EBL spans from 8.5 to 23%, with an associated mortality rate of approximately 4.6% from variceal bleeding (18–20). Additionally, the actuarial probability of bleeding from GV over a median follow-up of 26 months was reported to be 13% in patients receiving ECI for primary prophylaxis (21). In contrast, the 2-year variceal bleeding rate in this study was 11.6% among patients receiving variceal embolization, with no occurrences of variceal bleeding-related mortality.

This study also demonstrated that variceal embolization is safe. Up to 20% of patients with cirrhosis are intolerant to NSBBs (22), with approximately 3.7% experiencing serious adverse reactions (23). Additionally, the use of NSBBs in patients with Child–Pugh class C liver function remains controversial. There is potential for lethal hemorrhage from recent post-procedure ulcers after EBL (2). ECI in patients with high-risk GV has 3% serious complications and 7% overall mortality (21). In this study, severe complications occurred in only 2.3% (1 out of 43) of the cases following variceal embolization, with no fatalities reported. These findings suggest that variceal embolization is comparable to the methods recommended by the current guidelines in terms of safety.

This study used an enhanced variceal embolization technique, contributing to its efficacy and safety. Variceal embolization encompasses all interventional procedures aimed at occluding varicose veins, primarily through retrograde or anterograde approaches. Retrograde procedures, such as balloon-occluded retrograde transvenous obliteration (BRTO), are limited to patients with spontaneous splenorenal shunts. In contrast, anterograde embolization, which includes balloon-occluded antegrade transvenous obliteration (BATO) and PTVE, applies to a broader range of varices. In this study, the anterograde embolization technique was selected for its versatility. We opted for a non-balloon-assisted embolization method, avoiding the complexities of maneuvering balloon catheters into tortuous varices, which can lead to procedural failure and increased complications associated with balloon catheter indwelling, as seen in classical BRTO. A variety of catheters, such as the Cobra, MIK, and microcatheters, were utilized for the successful catheterization of varices, achieving almost a 100% success rate. Instead of using sclerosing agents, which have been associated with serious complications such as hemolysis and renal impairment (24), this study used a combination of steel coils and cyanoacrylate tissue adhesives. This approach not only shortened the operation time but also minimized complications (25, 26). The steel coils reduced blood flow, while the cyanoacrylate ensured complete and reticular embolization, effectively preventing ectopic embolization without the need for a balloon catheter post-operation. Cyanoacrylate was injected into an extensive variceal network, targeting the lower esophageal, periesophageal, paraesophageal, gastric cardiac, and perforating veins. This comprehensive and occlusive embolization strategy significantly reduced the likelihood of a short-term recurrence of EGV.

This study is subject to certain limitations. First, the study is retrospective in nature even though the data were collected prospectively. The findings require validation through prospective, large-sample studies. Second, the determination of when endoscopic treatments were difficult was subjective. Since both EBL and ECI require specific expertise, and because the proficiency of endoscopists can vary widely, the decision to prevent variceal bleeding is often influenced by the level of available local expertise at a given center. To minimize these limitations, particularly those associated with technical proficiency and interpretive variability, decisions were made collaboratively by two experienced endoscopists. This approach aimed to ensure a more standardized assessment and reduce the potential bias in determining the suitability of endoscopic treatment options.

In conclusion, the results of this study indicate that variceal embolization is a viable and safe alternative to the established standard treatments for the primary prophylaxis of variceal bleeding. This technique offers a definitive treatment pathway for patients who are intolerant to NSBBs or endoscopic therapies.

## Data availability statement

The original contributions presented in the study are included in the article/supplementary material, further inquiries can be directed to the corresponding authors.

## Ethics statement

The studies involving humans were approved by the Medical Ethics Committee of the First Affiliated Hospital of Air Force Medical University. The studies were conducted in accordance with the local legislation and institutional requirements. The participants provided their written informed consent to participate in this study.

## Author contributions

JT: Conceptualization, Data curation, Formal analysis, Funding acquisition, Investigation, Methodology, Project administration, Resources, Software, Supervision, Validation, Visualization, Writing – original draft, Writing – review & editing. XY: Methodology, Data curation, Resources, Validation, Visualization, Writing – review & editing. YZ: Investigation, Methodology, Resources, Validation, Writing – review & editing. KL: Data curation, Formal analysis, Investigation, Methodology, Software, Writing – review & editing. XG: Resources, Writing – review & editing. NH: Data curation, Resources, Software, Validation, Visualization, Writing – review & editing. JN: Data curation, Investigation, Methodology, Resources, Validation, Visualization, Writing – review & editing. JX: Resources, Validation, Visualization, Writing – review & editing. WW: Software, Validation, Writing – review & editing. YS: Software, Supervision, Validation, Visualization, Writing – review & editing.
